# A comparison of myocardial magnetic resonance extracellular volume mapping at 3 T against histology of tissue collagen in severe aortic valve stenosis and obstructive hypertrophic cardiomyopathy

**DOI:** 10.1007/s10334-023-01070-6

**Published:** 2023-02-23

**Authors:** Adrianus J. Bakermans, Merel Kouwenhoven, Judith de Vos, Dylan K. de Vries, Yolan J. Reckman, Emile S. Farag, David R. Koolbergen, Jolanda Kluin, Aart J. Nederveen, Gustav J. Strijkers, S. Matthijs Boekholdt

**Affiliations:** 1 Department of Radiology and Nuclear Medicine, Amsterdam University Medical Centers, University of Amsterdam, Amsterdam, The Netherlands; 2 Biomedical Engineering and Physics, Amsterdam University Medical Centers, University of Amsterdam, Amsterdam, The Netherlands; 3 Department of Experimental Cardiology, Amsterdam University Medical Centers, University of Amsterdam, Amsterdam, The Netherlands; 4 Department of Cardiothoracic Surgery, Amsterdam University Medical Centers, University of Amsterdam, Amsterdam, The Netherlands; 5 Department of Cardiology, Amsterdam University Medical Centers, University of Amsterdam, Amsterdam, The Netherlands

**Keywords:** Histology, Myocardial fibrosis, Native T_1_ mapping, Picrosirius red, Quantitative magnetic resonance imaging

## Abstract

**Objective:**

Quantitative extracellular volume fraction (ECV) mapping with MRI is commonly used to investigate in vivo diffuse myocardial fibrosis. This study aimed to validate ECV measurements against ex vivo histology of myocardial tissue samples from patients with aortic valve stenosis or hypertrophic cardiomyopathy.

**Materials and methods:**

Sixteen patients underwent MRI examination at 3 T to acquire native T_1_ maps and post-contrast T_1_ maps after gadobutrol administration, from which hematocrit-corrected ECV maps were estimated. Intra-operatively obtained myocardial tissue samples from the same patients were stained with picrosirius red for quantitative histology of myocardial interstitial fibrosis. Correlations between in vivo ECV and ex vivo myocardial collagen content were evaluated with regression analyses.

**Results:**

Septal ECV was 30.3% ± 4.6% and correlated strongly (*n* = 16, *r* = 0.70; *p* = 0.003) with myocardial collagen content. Myocardial native T_1_ values (1206 ± 36 ms) did not correlate with septal ECV (*r* = 0.41; *p* = 0.111) or with myocardial collagen content (*r* = 0.32; *p* = 0.227).

**Discussion:**

We compared myocardial ECV mapping at 3 T against ex vivo histology of myocardial collagen content, adding evidence to the notion that ECV mapping is a surrogate marker for in vivo diffuse myocardial fibrosis.

## Introduction

Quantitative magnetic resonance imaging (MRI) techniques have become an established diagnostic imaging modality to noninvasively investigate myocardial tissue characteristics. The spatial mapping of myocardial T_1_ relaxation time constants can be clinically relevant in diseases where myocardial interstitial fibrosis may play a role, such as valvular disease and cardiomyopathies [1, 2]. Whereas *focal* fibrotic areas due to scarring (e.g., ischemic injury) with consequentially large spatial variations are typically visualized through late gadolinium enhancement (LGE) MRI, *diffuse* interstitial fibrosis with a global and relatively homogeneous distribution cannot be detected with LGE MRI [3]. Yet, after the administration of a gadolinium-based contrast agent, quantitative T_1_ mapping allows for the calculation of extracellular volume fraction (ECV) maps and is particularly sensitive to the interstitial volume expansion that occurs with fibrosis. However, both native T_1_ as well as ECV mapping do not directly detect tissue collagen or extracellular matrix components, and therefore provide surrogate markers of myocardial fibrosis.

Although it was recognized already in the mid-1980s that proton nuclear magnetic resonance relaxation times are related to the presence of collagen fibers in histological assays of human myocardial tissue samples [4], direct comparisons of in vivo quantitative MRI against ex vivo histology of myocardial fibrosis were not reported until more than 20 years later [5, 6]. Most of the initial validation studies were conducted at a magnetic field strength of 1.5 T, with only some of the more recent work conducted on 3 T systems (Table 1). Histological assays of myocardial fibrosis require access to tissue, which is challenging to obtain from the in vivo human heart. This likely explains the paucity of data that is available on histological validation of MRI-measured ECV, particularly at 3 T. Indeed, such direct comparisons have only been reported for intra-operative left ventricular septal biopsies from patients with valve disease [7], or for right ventricular septal biopsies during right ventricular catheterization of patients with dilated cardiomyopathy [8] and patients with heart failure with preserved ejection fraction (HFpEF) [9].

**Table 1:** Literature reports on histological validation of myocardial fibrosis measurements by magnetic resonance imaging (MRI) mapping of myocardial T_1_ relaxation time constants and extracellular volume fractions (ECV) in the human heart

	Year	Sequence	B_0_ (T)	Regression analyses			Staining	Condition
				*n*	*r*	*p*		
Native T_1_								
Bull et al. [15]	2013	ShMOLLI	1.5	19	0.65	0.002	PSR	AS
Miller et al. [16]	2013	MOLLI	1.5	6	0.20	0.71	PSR	Heart transplantation
Lee et al. [24]	2015	MOLLI	3	20	0.78	< 0.001	PSR	AS
de Meester de Ravenstein et al. [7]	2015	MOLLI	3	31	− 0.18^a^	0.32^a^	PSR	Valve diseases
Lurz et al. [19]	2016	MOLLI	1.5/3	77	NR^b^	NR^b^	PSR	Myocarditis
Nakamori et al. [8]	2018	MOLLI	3	36	0.77	< 0.001	PSR	DCM
Nakamori et al. [8]	2018	MOLLI	3	36	0.55^c^	< 0.001	H&E	DCM
Park et al. [25]	2019	MOLLI	1.5	71	0.43	0.0002	MT	AS
Omori et al. [9]	2020	MOLLI	3	19	0.44	0.06	PSR	HFpEF
Balčiūnaitė et al. [26]	2022	MOLLI	1.5	67	NR	NS	MT	AS
* This work*	2023	MOLLI	3	8	0.36	0.38	PSR	AS
* This work*	2023	MOLLI	3	8	0.29	0.49	PSR	HCM
* This work*	2023	MOLLI	3	16	0.32	0.23	PSR	AS and HCM
Post-contrast T_1_								
Iles et al. [5]	2008	FLASH-IR	1.5	9	− 0.70	0.03	PSR	Heart transplantation
Sibley et al. [27]	2012	Look-Locker-IR	1.5	47	− 0.57	< 0.0001	MT	Cardiomyopathy
White et al. [28]	2013	ShMOLLI	1.5	18	− 0.46	0.04	PSR	AS
Miller et al. [16]	2013	MOLLI	1.5	6	− 0.21	0.69	PSR	Heart transplantation
Mascherbauer et al. [17]	2013	FLASH-IR	1.5	9	− 0.98	< 0.01	mTCR	HFpEF
Ellims et al. [18]	2014	FLASH-IR	1.5	9	− 0.70	0.03	PSR	HCM
Iles et al. [29]	2015	FLASH-IR	1.5	12	− 0.78	0.003	MT	HCM and heart transplantation
de Meester de Ravenstein et al. [7]	2015	MOLLI	3	31	− 0.36	0.05	PSR	Valve diseases
* This work*	2023	MOLLI	3	8	− 0.30	0.47	PSR	AS
* This work*	2023	MOLLI	3	8	− 0.09	0.83	PSR	HCM
* This work*	2023	MOLLI	3	16	− 0.20	0.46	PSR	AS and HCM
ECV								
Flett et al. [6]	2010	FLASH-IR	1.5	18	0.93	< 0.001	PSR	AS
Flett et al. [6]	2010	FLASH-IR	1.5	8	0.79	0.08	PSR	HCM
Flett et al. [6]	2010	FLASH-IR	1.5	26	0.89	< 0.001	PSR	AS and HCM
Fontana et al. [30]	2012	FLASH-IR	1.5	18	0.77	NR	PSR	AS
Fontana et al. [30]	2012	ShMOLLI	1.5	18	0.83	NR	PSR	AS
White et al. [28]	2013	ShMOLLI	1.5	18	0.83	< 0.01	PSR	AS
Miller et al. [16]	2013	MOLLI	1.5	6	0.94	0.004	PSR	Heart transplantation
Aus dem Siepen et al. [20]	2015	MOLLI	1.5	24	0.85	0.01	AFOG	DCM
de Meester de Ravenstein et al. [7]	2015	MOLLI	3	31	0.78	< 0.001	PSR	Valve diseases
Kammerlander et al. [31]	2016	MOLLI	1.5	36	0.49	0.003	mT	Heart failure and valve diseases
Treibel et al. [32]	2016	ShMOLLI	1.5	18	0.83	< 0.001	PSR	AS
Lurz et al. [19]	2016	MOLLI	1.5/3	77	NR^b^	NR^b^	MT	Myocarditis
Nakamori et al. [8]	2018	MOLLI	3	36	0.66	< 0.001	PSR	DCM
Nakamori et al. [8]	2018	MOLLI	3	36	0.86^c^	< 0.001	H&E	DCM
Treibel et al. [33]	2018	MOLLI	1.5	133	NR	NS	PSR	AS
Park et al. [25]	2019	MOLLI	1.5	71	0.47	< 0.0001	MT	AS
Omori et al. [9]	2020	MOLLI	3	19	0.54	0.02	PSR	HFpEF
Pucci et al. [22]	2021	MOLLI	1.5	20	NR	0.33	MT	Amyloidosis
Balčiūnaitė et al. [26]	2022	MOLLI	1.5	67	NR	NS	MT	AS
* This work*	2023	MOLLI	3	8	0.77	0.03	PSR	AS
* This work*	2023	MOLLI	3	8	0.57	0.14	PSR	HCM
* This work*	2023	MOLLI	3	16	0.70	0.003	PSR	AS and HCM

*AFOG* acid fuchsin orange G, *AS* aortic valve stenosis, *DCM* dilated cardiomyopathy, *ECV* extracellular volume fraction, *HCM* hypertrophic cardiomyopathy, *HFpEF* heart failure with preserved ejection fraction, *H&E* hematoxylin and eosin, *MT* Masson’s trichrome, *mTCR* modified trichrome and Congo red, *PSR* picrosirius red, *NR* not reported, *NS* not significant

^a^As reported in Figure and Abstract; main text reports *r* = − 0.15, *p* = 0.41

^b^Validation against histopathological diagnosis

^c^Correlation with histological extracellular space component

Here, we report the validation of quantitative MRI of diffuse myocardial fibrosis at 3 T in patients with severe aortic valve stenosis or obstructive hypertrophic cardiomyopathy (HCM), where biopsies or myectomy samples for histological assays could be intra-operatively obtained from the left ventricular septum, respectively. We hypothesized that septal ECV, estimated with in vivo measurements of native and post-contrast myocardial and blood T_1_ relaxation time constants, would correlate with quantitative histological assays of myocardial collagen content in tissue obtained from the same patients.

## Materials and methods

The study was approved by the local institutional review board (NL52084.018.15; Academic Medical Center, Amsterdam, The Netherlands). All participants provided written informed consent. This work was conducted within the framework of a study on the validation of quantitative MR of the human heart at 3 T, of which results on proton MR spectroscopy measurements have been published previously [10].

### Subjects

We prospectively recruited 26 patients with a clinical indication for open-heart surgery at the Departments of Cardiology and Cardiothoracic Surgery at the Amsterdam University Medical Centers, Amsterdam, The Netherlands. Patients were eligible if they were scheduled for surgical aortic valve replacement in case of severe aortic valve stenosis, or for septal myectomy in case of obstructive HCM. Both procedures allow access to the left ventricular septal myocardium that can be exploited for the collection of myocardial tissue samples, without additional risks for the patient. Exclusion criteria were an estimated glomerular filtration rate (eGFR) of < 30 mL/min/1.73 m^2^, the presence of any non-MR compatible implants, and other contraindications for MR examination at 3 T. All participants underwent the MR protocol as described below. Furthermore, venous blood samples were collected in 4-mL EDTA tubes just prior to MR examination through the intravenous cannula that was inserted in the median cubital vein for contrast agent administration. Hematocrit was determined immediately after sample collection according to standard laboratory assays. Within 1 week after MR examination, myocardial tissue samples from the left ventricular septum were collected intra-operatively, either with a Tru-cut biopsy needle during surgical aortic valve replacement, or through septal myectomy in HCM. Any layer of endocardial fibrotic tissue (e.g., fibroelastosis) was removed, and tissue samples were immediately stored in pH = 7 buffered 3.6% m/v formaldehyde solution (Orphi Farma BV, Lage Zwaluwe, The Netherlands) until processing for histology.

### MR protocol

All MR examinations were performed with a 3 T MR system (Ingenia; Philips, Best, The Netherlands) equipped with a 16-channel anterior receiver coil array and a 12-channel posterior receiver coil array integrated in the patient bed. Subjects were positioned supine and connected to a 4-lead ECG sensor. As part of a comprehensive protocol [10], native T_1_ maps were acquired at end-diastole using a balanced steady-state free-precession gradient-echo modified Look-Locker inversion-recovery (MOLLI) sequence [11] according to a 5 s (3 s) 3 s sampling scheme [12]. Imaging parameters: field of view, 320 × 380 mm; slice thickness, 8 mm; repetition time, 2.6 ms; flip angle, 35°; matrix, 174 × 192; parallel imaging (SENSE) factor, 4; inversion times, 140–5400 ms. Quantitative T_1_ maps of the left ventricular long-axis four-chamber view (*n* = 16), the basal (*n* = 16) and mid-ventricular (*n* = 12) short-axis views [1] were acquired during an end-expiration breath hold per slice. Then, a bolus of 0.1 mmol/kg body weight gadobutrol (1.0 mmol/mL Gd-DO3A-butrol, Gadovist; Bayer AG, Leverkusen, Germany) was administered via a cannula in the median cubital vein followed by a flush of 15 mL of normal saline. After 10—20 min, post-contrast T_1_ maps in geometries and orientations that matched the native T_1_ map views were acquired using a MOLLI sequence according to a 4 s (1 s) 3 s (1 s) 2 s sampling scheme and inversion times 140–4500 ms, with otherwise identical imaging parameters. All inversion-recovery image series were stored and processed offline to generate native and post-contrast T_1_ maps.

### MR data analyses

**Fig. 1:**
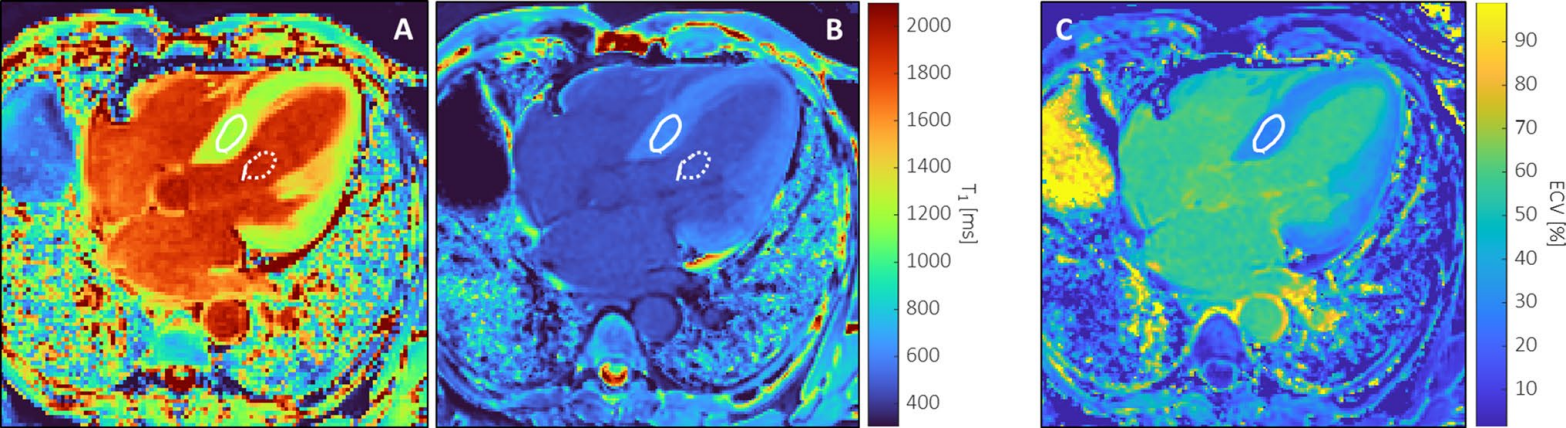
Myocardial T_1_ and ECV mapping at 3 T. A four-chamber native T_1_ map (**A**) was acquired with a 5 s (3 s) 3 s MOLLI sequence, while 12 min after administration of 0.1 mmol/kg body weight gadobutrol a 4 s (1 s) 3 s (1 s) 2 s MOLLI sequence was used (**B**). The extracellular volume fraction (ECV; **C**) was calculated using blood T_1_ estimated from a region of interest (ROI) drawn in the left ventricular blood pool (dotted line) and blood hematocrit determined in a venous blood sample obtained just prior to MR examination. This case is a 61-year-old male (body mass index, 25.8 kg/m^2^) with obstructive hypertrophic cardiomyopathy scheduled for septal myectomy. Native T_1_ in the septal ROI (solid line) was 1206 ± 29 ms, and 1933 ± 30 ms for the blood pool. After contrast agent administration, T_1_ values were 615 ± 16 ms and 474 ± 12 ms, respectively. With hematocrit at 0.43 L/L, septal ECV was calculated at 28.1% ± 1.9%

The quantitative evaluation of the MOLLI image series was performed with QMap 2.2 (Medis medical imaging systems BV, Leiden, The Netherlands) according to consensus recommendations [13]. Per series, any in-plane respiration-induced offsets between images were corrected with rigid translations. Likewise, any in-plane shifts between native and post-contrast T_1_ maps were corrected. Regions of interest (ROI) in the septum were drawn manually on the long-axis four-chamber and the short-axis views [1, 13], conservatively avoiding voxels at the endocardial borders that may suffer from partial volume effects with blood from either the left or right ventricular lumen (Fig. 1). Focal regions of low myocardial post-contrast T_1_ values and high ECV that would reflect LGE-positive areas were not included in these homogeneous ROIs, effectively excluding any focal myocardial fibrosis from the analyses. A second ROI was defined in the left ventricular blood pool, avoiding the inclusion of papillary muscles. Myocardial and left ventricular blood T_1_ relaxation time constants were estimated by voxel-wise fitting of an exponential curve through the signal intensities within the ROIs for the inversion-recovery series. ECV [%] was calculated in a voxel-wise fashion according to

$${\text{ECV}} = (1 - {\text{Hct}}) \times \left[ {{\raise0.7ex\hbox{${\left( {{\text{R}}_{{1,\,_{{\text{myocardium post}}} }} - {\text{R}}_{{1,\,_{{\text{myocardium native}}} }} } \right)}$} \!\mathord{\left/ {\vphantom {{\left( {{\text{R}}_{{1,\,_{{\text{myocardium post}}} }} - {\text{R}}_{{1,\,_{{\text{myocardium native}}} }} } \right)} {\left( {{\text{R}}_{{1,\,_{{\text{blood post}}} }} - {\text{R}}_{{1,\,_{{\text{blood native}}} }} } \right)}}}\right.\kern-0pt} \!\lower0.7ex\hbox{${\left( {{\text{R}}_{{1,\,_{{\text{blood post}}} }} - {\text{R}}_{{1,\,_{{\text{blood native}}} }} } \right)}$}}} \right] \times 100,$$

with Hct the blood hematocrit [L/L] at the time of MR examination, and R_1_ the respective relaxation rates (i.e., 1/T_1_ in ms^−1^) of myocardium and blood measured before (native) and after (post) contrast agent administration, respectively. Quantifications were averaged over the ROIs to yield mean native and post-contrast T_1_ values and ECV per subject.

### Histology

**Fig. 2:**
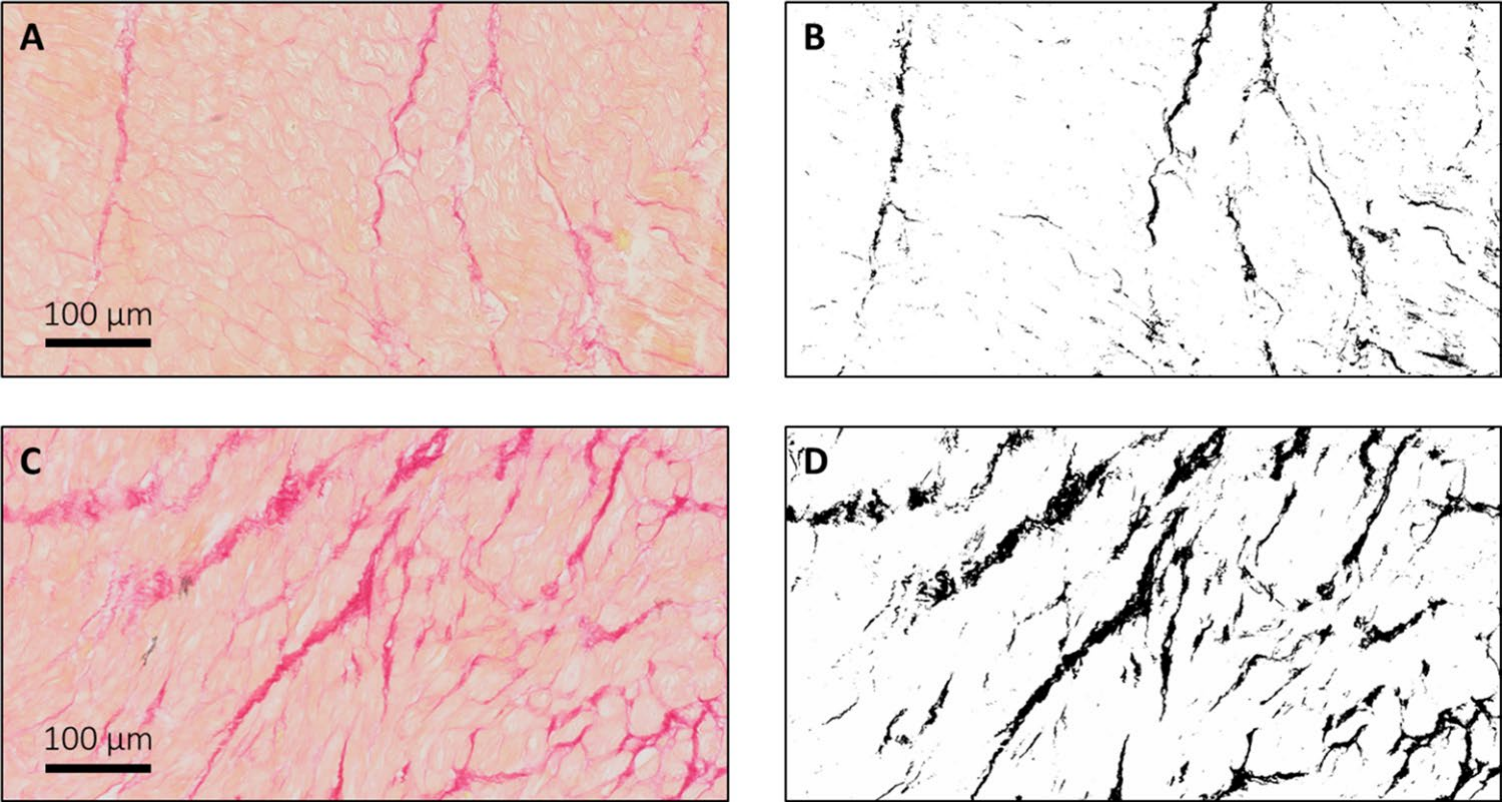
Histology of myocardial interstitial fibrosis. Images of picrosirius red-stained myocardial tissue samples were obtained at 40 × magnification (**A**, **C**), from which the myocardial collagen content was determined by isolating the picrosirius red-stained surface area (**B**, **D**) from the original images. Myocardial collagen content in these sections was 3.2% (**A**, **B**) and 9.2% (**C**, **D**)

Myocardial tissue samples from the left ventricular septum were embedded in paraffin, sectioned at 3-µm slices, and stained all at once with picrosirius red to detect myocardial collagen. Stained sections were digitized with a IntelliSite Ultra Fast Scanner 1.6 (Philips, The Netherlands) at 40 × magnification. From these digitized slides, six non-overlapping digital images were grabbed for each specimen. Endocardial and perivascular areas were avoided or excluded from the analyses. Images were loaded into ImageJ (v1.53; National Institutes of Health, Bethesda, MD), and minimum and maximum signal intensities for picrosirius red-stained collagen were estimated by drawing ROIs in areas of interstitial fibrosis. The resultant signal intensity bandwidth was used to isolate picrosirius red-stained surfaces in all images (Fig. 2). A similar approach was used to identify and exclude any background signal from the myocardial surface area. Myocardial collagen content was expressed as a percentage by dividing the picrosirius red-stained surface area by the total myocardial surface area. Per subject, myocardial collagen content in six images was averaged to yield a quantitative estimate of myocardial interstitial fibrosis.

### Statistical analyses

Data are presented as mean ± standard deviation (SD). Regression analyses between myocardial native T_1_ and ECV and histological quantifications of the myocardial collagen content were conducted using MATLAB (The MathWorks, Inc., Natick, MA, USA). Specifically, a relative equal weighting of the errors between the linear fit and the in vivo MR measurements and ex vivo histological assays was assumed. The covariance matrices for the slope and intercept parameters were determined as described previously [14], and used to determine the 95% confidence intervals of the linear fit. The level of significance was set at *p* < 0.05.

## Results

Out of the 26 recruited patients, three patients experienced shortness-of-breath, dizziness or claustrophobia during MR examination, in which cases the session was aborted before the acquisition of quantitative MRI data. No contrast agent could be administered to three patients due to the unavailability of a qualified research nurse. Myocardial tissue samples were not collected for these cases. Biopsies were unavailable for one patient undergoing aortic valve replacement due to insufficient access to the septum during surgery. An insufficient amount of tissue was obtained in another three patients, which prevented histological evaluation. Thus, myocardial tissue samples of 16 patients (*n* = 8 with aortic valve stenosis, *n* = 8 with obstructive HCM; male/female, 11/5; age, 65.9 ± 8.6 years; body mass index, 26.8 ± 5.2 kg/m^2^) who completed the MR examination prior to surgery were collected intra-operatively, allowing a direct comparison of quantitative in vivo MRI readouts against ex vivo histology in 16 cases.

**Fig. 3:**
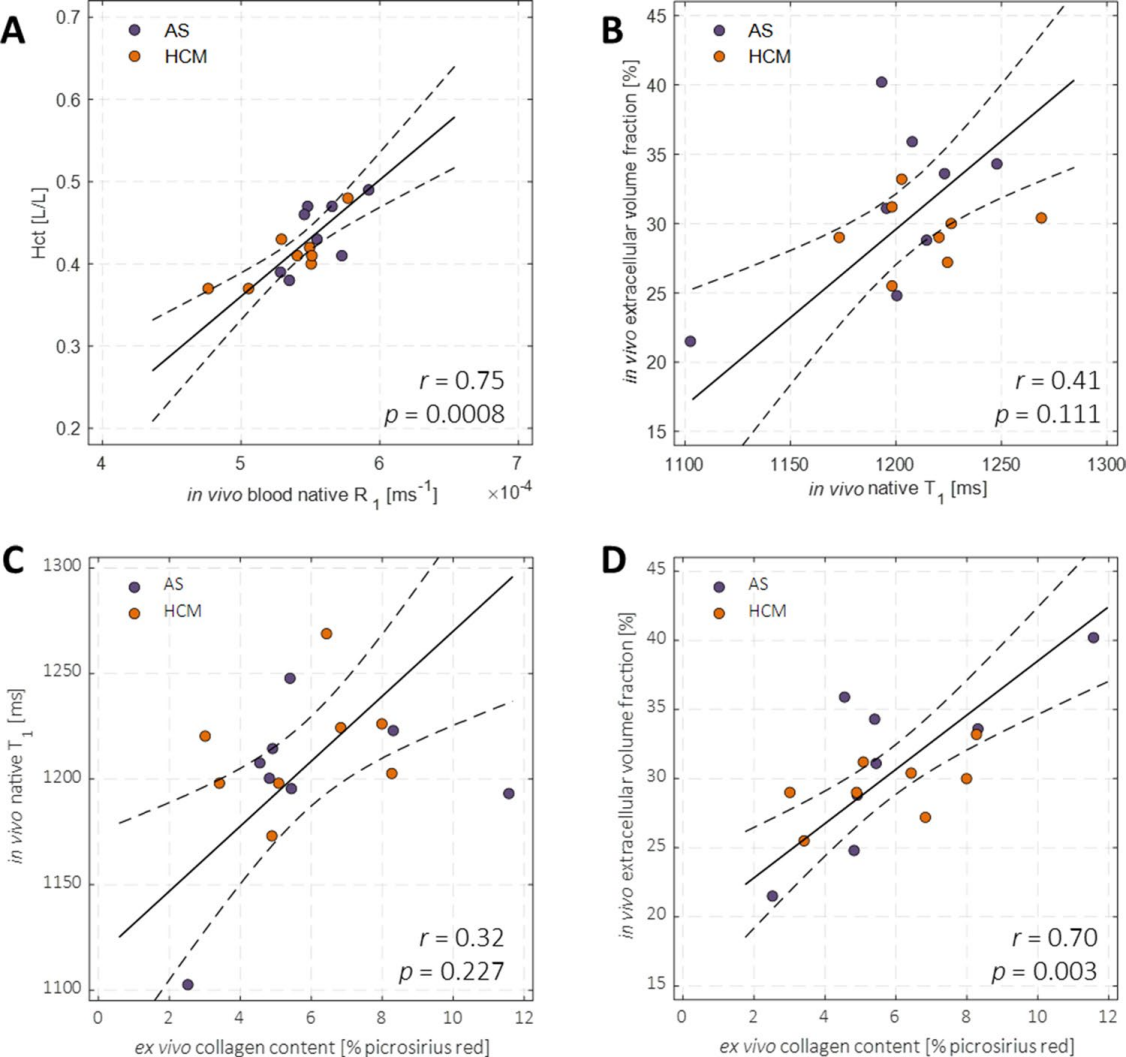
Regression analyses of the native longitudinal relaxation rate R_1_ of left ventricular blood with blood hematocrit (**A**), and of in vivo myocardial native T_1_ and extracellular volume fraction (ECV) measured with quantitative magnetic resonance imaging (MRI) at 3 T (**B**) against ex vivo myocardial collagen content determined histologically with picrosirius red staining of myocardial tissue samples obtained from the same patients (**C**, **D**, respectively). Dashed lines indicate the 95% confidence intervals of the linear model fits (solid lines). *AS* aortic valve stenosis, *HCM* hypertrophic cardiomyopathy, *Hct* hematocrit

Myocardial native T_1_ values were 1206 ± 36 ms, and were similar for aortic valve stenosis and HCM patients (1198 ± 42 vs. 1214 ± 28 ms; *p* = 0.394). Left ventricular blood native T_1_ was 1839 ± 98 ms, and R_1,blood native_ correlated strongly (*n* = 16, *r* = 0.75; *p* < 0.001; Fig. 3A) with blood hematocrit (0.42 ± 0.04 L/L). Yet, R_1,blood native_ nor blood hematocrit correlated with myocardial native T_1_, indicating that blood within the myocardium did not confound myocardial native T_1_ measurements. Septal ECV was 30.3% ± 4.6%, and was similar for aortic valve stenosis and HCM patients (31.3% ± 6.1% vs. 29.4% ± 2.4%; *p* = 0.436). Myocardial collagen content, expressed as the percentage of picrosirius red-stained surface area, was 5.8 ± 2.3%, and was similar for aortic valve stenosis and HCM patients (5.9% ± 2.8% vs. 5.7% ± 2.0%; *p* = 0.870). Notably, myocardial native T_1_ values did not correlate with septal ECV (*r* = 0.41; *p* = 0.111; Fig. 3B) or with myocardial collagen content (*r* = 0.32; *p* = 0.227; Fig. 3C, Table 1). Likewise, myocardial post-contrast T_1_ values (574 ± 82 ms) did not correlate with myocardial collagen content (*r* = − 0.20; *p* = 0.462; Table 1). Importantly, septal ECV correlated strongly with myocardial collagen content (*n* = 16, *r* = 0.70; *p* = 0.003; Fig. 3D, Table 1), particularly in aortic valve stenosis patients (*n* = 8, *r* = 0.77; *p* = 0.025; Table 1), but not significantly in HCM patients (*n* = 8, *r* = 0.57; *p* = 0.14; Table 1).

## Discussion

This work demonstrates that myocardial ECV mapping at 3 T can quantify diffuse myocardial fibrosis, evidenced by a strong correlation between in vivo MRI measurements and ex vivo histology of myocardial interstitial fibrosis. Moreover, we show that the quantification of myocardial native T_1_ appears to be insufficient for estimating the degree of diffuse myocardial fibrosis, but that concomitant T_1_ maps acquired after the administration of a gadolinium-based contrast agent are required to probe the interstitial volume. Our data in patients with aortic valve stenosis and obstructive HCM corroborate and expand the validations of myocardial ECV mapping at 3 T as a surrogate marker of myocardial collagen content that were reported for patients with valve diseases [7], dilated cardiomyopathy [8], and HFpEF [9].

In contrast to some previous studies that reported a correlation between myocardial native T_1_ values and histological assays of tissue collagen content with picrosirius red staining, we could not establish such a relation. Correlations in previous studies may have been influenced by subjects with high levels of fibrosis [8, 15]. Moreover, in studies with relatively small cohorts of *n* < 20 subjects such as in the present work, myocardial native T_1_ did not correlate with tissue collagen content [9, 16]. Together, this suggests that the validity of native T_1_ mapping as a sensitive surrogate marker for diffuse myocardial fibrosis in individual patients may be limited. Indeed, other than myocardial fibrosis, native T_1_ increases with edema and amyloidosis, while sphingolipid accumulation in Fabry’s disease, fat infiltration, and iron overload can markedly decrease myocardial native T_1_ values [1]. Changes in native T_1_ are therefore not specific to alterations in tissue collagen content. When probing the interstitial volume specifically through the administration of a gadolinium-based contrast agent in combination with ECV mapping, the sensitivity of quantitative MRI to myocardial fibrosis greatly improves. This has been demonstrated by strong correlations between myocardial ECV and quantitative histological assays at different magnetic field strengths and for various stainings of tissue collagen (Table 1), even in a study with a cohort of only *n* = 6 patients [16].

Some well-controlled studies report a conceptually consistent negative correlation between myocardial post-contrast T_1_ values and tissue collagen content, even in small cohorts of *n* < 10 subjects [5, 17, 18]. Myocardial post-contrast T_1_ values are susceptible to physiological (e.g., blood pool size, renal clearance rate) and experimental (e.g., acquisition delay after contrast agent administration [16]) variations that can typically not be fully controlled for in a clinical setting. Myocardial ECV mapping by a combination of native T_1_ mapping and post-contrast T_1_ mapping separated by a delay of 10–30 min, in conjunction with concomitant blood sampling for an estimate of current hematocrit, mitigates most of these effects [1].

Quantitative ECV mapping remains a *surrogate* evaluation of diffuse myocardial fibrosis. Here, we established a good agreement between in vivo MRI measurements and ex vivo tissue collagen content in a direct comparison in patients with severe aortic valve stenosis and obstructive HCM. Although other work has demonstrated similar results in other diseases such as myocarditis [19], dilated cardiomyopathy [8, 20], and HFpEF [9], caution is warranted when extrapolating ECV mapping as a surrogate readout for myocardial fibrosis to other conditions or diseases. We found previously that myocardial ECV, but not native T_1_, transiently increases directly after marathon running in trained athletes [21], which is suggestive of myocardial interstitial edema induced by prolonged high-intensity exercise. Indeed, the acute and transient nature of such an ECV increase argues against the presence of collagen fibers and the development of myocardial fibrosis, but rather reflects a temporary expansion of the interstitial volume. In amyloidosis, a condition where misfolded proteins are deposited extracellularly, it was shown that MRI-measured ECV correlated with Congo red-stained amyloid deposits and even stronger with the sum of amyloid and Masson’s trichrome-stained interstitial fibrosis (*n* = 20, *r* = 0.66; *p* = 0.001), but importantly, not with fibrosis alone [22]. Those studies emphasize that detected changes or differences in myocardial ECV do not necessarily reflect alterations in myocardial collagen content specifically.

We report a stronger correlation between ECV and myocardial collagen content in aortic valve stenosis than in obstructive HCM, which is strikingly similar to what was previously found in such patients at 1.5 T [6]. Myocardial ECV mapping has not been extensively performed in HCM, particularly not at 3 T (Table 1). The phenotype of HCM is highly variable [18], and studies in larger cohorts of patients with HCM are warranted to corroborate our findings. Our relatively small cohorts for both aortic valve stenosis as well as HCM prevent the extrapolation of our findings to a generic interpretation of in vivo myocardial fibrosis in these conditions. Fibrosis may occur less homogeneously in HCM than in aortic valve stenosis, e.g., in the form of myocardial disarray, regional replacement fibrosis, and diffuse interstitial fibrosis [23], which increases the risk of potential sampling errors relative to the large ROIs that are used to estimate septal ECV according to the consensus recommendations [1, 13] followed here and elsewhere [6]. Additionally, the ranges of ECV values and myocardial collagen content values were narrower in HCM than in aortic valve stenosis, decreasing the sensitivity to establish a strong correlation in our cohort of HCM patients.

In conclusion, we have provided a direct comparison of myocardial ECV mapping at 3 T in patients with severe aortic valve stenosis and HCM against ex vivo quantitative histological assays of myocardial collagen content. Such validations contribute further evidence to the notion that MRI-measured ECV at 3 T is a surrogate marker for in vivo diffuse myocardial fibrosis. Yet, this approach may not hold in conditions or diseases where mechanisms other than myocardial collagen deposition leads to an expansion of the interstitial volume.

## Data Availability

The imaging data that support the findings of this study are available from the corresponding author upon reasonable request.
